# Hydrothermal enrichment of lithium in intracaldera illite-bearing claystones

**DOI:** 10.1126/sciadv.adh8183

**Published:** 2023-08-30

**Authors:** Thomas R. Benson, Matthew A. Coble, John H. Dilles

**Affiliations:** ^1^ Lithium Americas Corporation, Vancouver, Canada.; ^2^ Lamont-Doherty Earth Observatory, Columbia University, Palisades, NY, USA.; ^3^ GNS Science, Lower Hutt, New Zealand.; ^4^ College of Earth, Ocean and Atmospheric Sciences, Oregon State University, Corvallis, Oregon, USA.

## Abstract

Developing a sustainable supply chain for the global proliferation of lithium ion batteries in electric vehicles and grid storage necessitates the extraction of lithium resources that minimize local environmental impacts. Volcano sedimentary lithium resources have the potential to meet this requirement, as they tend to be shallow, high-tonnage deposits with low waste:ore strip ratios. Illite-bearing Miocene lacustrine sediments within the southern portion of McDermitt caldera (USA) at Thacker Pass contain extremely high lithium grades (up to ~1 weight % of Li), more than double the whole-rock concentration of lithium in smectite-rich claystones in the caldera and other known claystone lithium resources globally (<0.4 weight % of Li). Illite concentrations measured in situ range from ~1.3 to 2.4 weight % of Li within fluorine-rich illitic claystones. The unique lithium enrichment of illite at Thacker Pass resulted from secondary lithium- and fluorine-bearing hydrothermal alteration of primary neoformed smectite-bearing sediments, a phenomenon not previously identified.

## Teaser

Post-caldera hydrothermal fluids altered smectite-bearing sediments to Li-rich illite, creating an exceptional lithium deposit.

## INTRODUCTION

Ubiquitous impacts of climate change are stimulating a global transition to lower-carbon energy technologies such as electric vehicles and power generation from wind turbines and solar panels. Because these technologies require lithium ion batteries for energy storage, there is an international “lithium rush” to identify additional lithium (Li) resources to supply the forecasted ~1 million metric ton (MT) of Li demand by 2040, an 8× increase from total global 2022 production (*1*, *2*). Developing a sustainable and diverse supply chain to meet lower-carbon energy and national security goals (*3*) requires mining the highest-grade domestic Li resources with the lowest waste:ore strip ratios to minimize both the volume of material extracted from the Earth, and the carbon footprint associated with transportation of Li chemicals to cathode and battery manufacturing facilities.

**Fig. 1.**
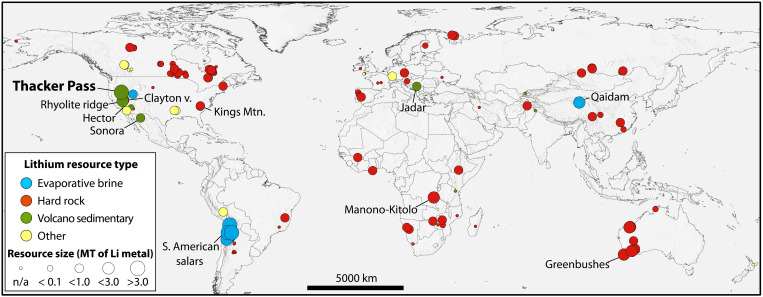
Map showing type and relative size of global lithium resources. Current production is predominantly spodumene from pegmatites in Australia (47%) and brines underlying salt flats in Chile (30%), China (12%), and Argentina (5%) (*2*). For more information, including resource data sources, see table S1.

Li resources are abundant globally, primarily occurring as pegmatites and greisen veins (hard rock) and high-elevation evaporitic brines (Fig. 1) and account for all current global Li production (*2*). A third type of Li resource, herein termed volcano sedimentary, occurs as sediments associated with, or adjacent to, centers of silicic volcanism. Chief among volcano sedimentary resources are the clay-rich lacustrine sediments deposited within the middle-Miocene McDermitt caldera, located along the Nevada-Oregon border on the northwestern edge of the Basin and Range province, USA (*4*–*9*). To date, 5.1 MT of Li have been reported as Canadian and Australian reporting code-compliant Measured and Indicated Resource estimates at the Lithium Americas Corp. Thacker Pass project (3.0 MT of Li) and the Jindalee Resources Ltd. McDermitt project (2.1 MT of Li) obtained through extensive drilling, resource, and economic modeling (*10*, *11*).

Recent calculations by Castor and Henry (*8*) estimate an in situ tonnage of ~20 to 40 MT of Li (maximum 120 MT of Li) to be contained within sediments of the whole McDermitt caldera. This back-of-the-envelope estimation is calculated using caldera-wide extrapolation of publicly available drill hole data from Lithium Americas Corp. and Jindalee Resources Ltd. and is not a reporting code-compliant mineral resource estimate that considers economic viability. Even if this estimation is high due to variations in sediment thickness and/or Li grade, the Li inventory contained in McDermitt caldera sediments would still be on par with, if not considerably larger than, the 10.2 MT of Li inventory estimated to be contained in brines beneath the Salar de Uyuni in Bolivia (*12*), previously considered the largest Li deposit on Earth.

The high tonnage of Li estimated to be contained in McDermitt caldera is due to the consistently high Li concentrations measured in lake sediments [>1000 parts per million (ppm) of Li] (*10*) relative to the average composition of terrestrial sedimentary rocks (<50 ppm) (*13*). The primary Li-bearing clay minerals are a trioctahedral magnesian smectite, similar to hectorite [Na_0.3_(Mg,Li)_3_Si_4_O_10_(OH)_2_] (*4*–*6*) and a trioctahedral illite similar to tainiolite [KMg_2_LiSi_4_O_10_(OH,F)_2_] (*14*); the latter yields the highest whole-rock concentrations of Li and has only been identified near the Thacker Pass project (*8*, *14*).

In this study, we analyzed smectite and illite clays within lacustrine sediments from a centralized zone enriched in Li, located in the southern resurgent portion of McDermitt caldera, near Thacker Pass. We present compositional data for Li, rubidium (Rb), boron (B), and other elements in Li-enriched illite clays measured in situ using sensitive high-resolution ion microprobe (SHRIMP-RG) to identify the degree and mechanisms of enrichment. These results are compared to caldera-wide Li assay data and geochemical and x-ray diffraction data from whole-rock samples and clay separates (*9*, *14*) to improve the metallogenic model of this deposit.

## RESULTS

### Geology and mineralogy of McDermitt caldera and intracaldera lacustrine sediments

The Li-rich clays discussed in this study are contained in caldera lake sediments of the ~40 × 30–km keyhole-shaped mid-Miocene McDermitt caldera. Volcanism at McDermitt caldera was contemporaneous with eruption of effusive Columbia River Basalt Group lavas between ~17 and 15 million years (Ma) ago (*15*–*17*) associated with Yellowstone plume volcanism (*18*). Mafic underplating and magmatic intrusions that fueled flood basalt volcanism caused low degrees of partial melting of continental lithosphere (*7*), forming peralkaline rhyolitic magma chambers in the upper crust (*19*, *20*). The high concentrations of Li in these magmas (averaging ~1400 ppm) is likely due to the pelitic composition of the assimilated metamorphic rocks (*21*–*24*) and further enrichment during fractional crystallization (*7*).

**Fig. 2.**
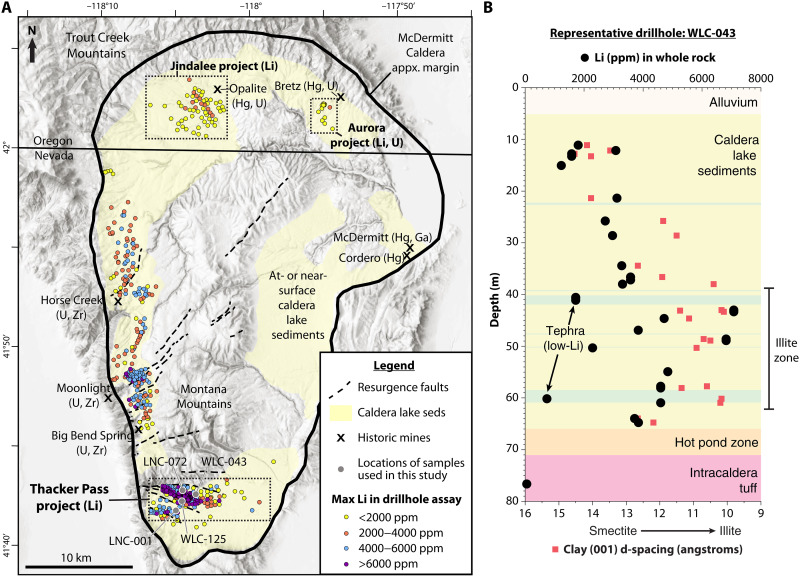
Lithium mineralization in McDermitt caldera. (**A**) Simplified map of McDermitt Caldera, locations of active and historic mining projects, locations of drillholes colored according to maximum downhole Li assay value, and locations of drillhole samples used in this study. Geology modified from (*20*). (**B**) Representative drillhole WLC-043 from the Thacker Pass project. Downhole whole-rock Li values (ppm) are shown in black circles and indicated on upper *x* axis. Clay (001) d-spacing values (angstroms) are shown in red squares and indicated on lower x-axis. Li concentrations increase with decreasing clay (001) d-spacing, demonstrating that the higher grades are associated with the illite clays. Drillhole data from (*8*, *10*, *11*, *28*, *29*) and previously unreleased Lithium Americas Corp. drilling.

At ~16.3 Ma, approximately 1000 km^3^ of Li-rich rhyolitic magma catastrophically evacuated in the eruption of the McDermitt Tuff, resulting in collapse of McDermitt caldera (*25*–*27*) (Fig. 2). About half the volume of the erupted material was emplaced as outflow ignimbrite up to 80 km from the caldera margin (*26*); the other half ponding within the caldera as thick (~1 km) intracaldera ignimbrite (*20*). Because the magma was peralkaline in composition, the tuff was emplaced at a higher temperature (>800°C) compared to most calc-alkaline rhyolite equivalents, leading to extensive welding, rheomorphism, and a prolonged degassing interval (*25*–*27*). The degassing intracaldera tuff (*9*) formed the floor of a massive paleo lake within the caldera, on which accumulated a thick (>200 m) sequence of tuffaceous lacustrine sediments until at least ~15.7 Ma ago (*8*). During this interval, intracaldera magmatic resurgence resulted in minor volcanism and uplift of the Montana Mountains (Fig. 2) in the southern portion of the caldera (*8*).

High concentrations of Li in lacustrine sediments at McDermitt caldera have been documented by several companies and researchers since the 1970s (*8*, *10*, *11*, *28*, *29*). The variably preserved volcanogenic intracaldera sediments cover ~70% of the ~1000-km^2^ McDermitt caldera (Fig. 2) and are locally more than 200 m thick (*8*, *10*). The sediments contain finely laminated claystone (60 to 90 volume percent) with subordinate lithologies of interbedded and variably altered volcanic tephra, rhyolitic tuff, tuffaceous siltstones and sandstones, carbonates, conglomerates, and mafic to felsic lavas (*28*). The dominant clay mineral identified in claystones throughout McDermitt caldera is magnesian smectite (*8*, *14*). At Thacker Pass, the smectites are similar to a trioctahedral hectorite with a (001) d-spacing between 12 and 14 Å (Fig. 2) (*8*, *14*). Other minerals present in the smectite-bearing claystones at Thacker Pass include calcite, dolomite, potassium feldspar, pyrite, analcime, and bitumen (*8*, *9*, *14*). At the Thacker Pass project, the dominant clay mineral ubiquitously transitions from magnesian smectite to a ~30 to 40-m-thick horizon dominated by trioctahedral Li-bearing illite with a (001) d-spacing of ~10 Å (Fig. 2) (*8*, *9*, *14*). This illite is most similar in composition to tainiolite based on its crystallography and high concentrations of Li, F, K, and Mg (*8*, *14*). Other phases identified in the illite-dominated portion of the sedimentary sequence include fluorite, K-feldspar, dolomite, pyrite, bitumen, and albite (*8*, *10*, *14*).

### Lithium enrichment in intracaldera claystones

Whole-rock assays from sedimentary intervals dominated by smectite clay throughout the caldera range, on average, from 1000 to 4000 ppm of Li (*10*, *11*), whereas illite intervals—only present at Thacker Pass—range from 4000 to 8000 ppm of Li (Fig. 2), with a maximum measured assay of ~9000 ppm of Li (*10*). Because these intentionally randomized assay intervals often include intercalated lithologies (e.g., tephra, carbonate, and basalt), the Li concentration of an assay interval is, in part, a function of the amount of nonclaystone rock included in the randomized material analysis. Hand-picked bulk claystone samples therefore represent a more accurate whole-rock claystone composition. Where analyzed, hand-picked bulk claystone geochemical analyses range from 2300 to 3700 ppm of Li in smectite-dominated intervals and 5700 to 8900 ppm of Li in illite-dominated intervals (Table 1) (*14*). Clay concentrates derived from these bulk samples via carbonate dissolution, and size fraction centrifugation have an average of ~5100 ppm of Li for Thacker Pass smectite and ~12,100 ppm of Li for illite at Thacker Pass (*9*, *14*). Thacker Pass illite samples contain more than twice the concentrations of Li measured using the same methodologies compared to other known smectite Li deposits (Table 1), suggesting that the illite at Thacker Pass formed in an unusual metallogenic environment.

**Table 1.** Average concentrations of Li (ppm) and K_2_O (wt %) in McDermitt caldera claystones and other known lithium-bearing claystones (*9*, *14*). nd, not determined.

Location	Clay type	Bulk rock		Clay concentrate	
		Li (ppm)	K_2_O (%)	Li (ppm)	K_2_O (%)
McDermitt caldera (NV)	Smectite	3130	1.95	5,060	1.52
McDermitt caldera (NV)	Illite	7250	6.46	12,050	7.28
Clayton valley (NV)	Illite/smectite	1250	5.59	3,430	4.28
Rhyolite ridge (NV)	Smectite	2410	0.63	6,770	0.12
Hector (CA)	Smectite	2780	0.17	5,710	0.12
Synthetic hectorite	Smectite	2880	nd	3,670	nd

### In situ measurement of lithium-bearing illites from Thacker Pass

**Fig. 3.**
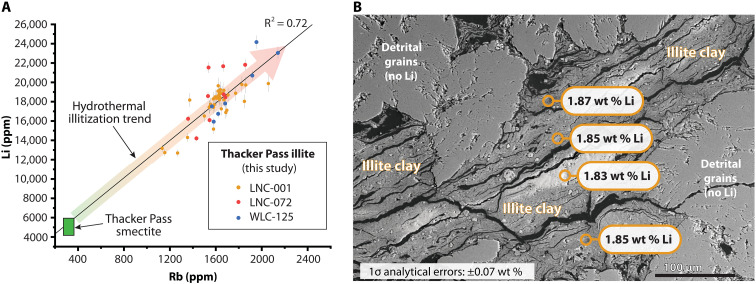
In situ SHRIMP-RG analysis of Li-illite at Thacker Pass. (**A**) Concentration of Li and Rb from three drillhole samples at Thacker Pass compared to composition of Thacker Pass smectite. The linear best-fit regression line through the illite data (black line) has a slope of 10.8 (equivalent to a Li:Rb molar ratio of ~120:1) and intersects the range of Li and Rb concentrations of clay concentrates from Thacker Pass smectite (*14*). (**B**) Merged cathodoluminescent backscatter image of illite from LNC-001 with locations of spot analyses shown. See the Supplementary Materials for full dataset (table S2).

To evaluate where Li is located spatially within bulk samples, the concentration of Li and other elements was measured in situ from the high-grade illite at Thacker Pass. Three samples of illite-bearing core were chosen for in situ analysis on SHRIMP-RG spanning the range of assay compositions in the deeper illitic section of the Thacker Pass deposit: 2760 ppm of Li (LNC-072), 5610 ppm of Li (LNC-001), and 7102 ppm of Li (WLC-125). Samples LNC-072 and WLC-125 are from the future Thacker Pass pit area, and sample LNC-001 is from south of St. Rt. 293 (Fig. 2). Samples were impregnated with epoxy and carefully polished to expose illite clay layers that were tens to hundreds of microns in thickness (Fig. 3). The high spatial resolution of this method (~12-μm-diameter spot, 1 to 2 μm deep) was critical for avoiding minor minerals such as calcite and dolomite that could not be successfully removed from bulk samples (*14*) and are not Li-bearing materials.

In situ Li concentrations from illite-bearing samples at Thacker Pass range in composition from ~1.3 to 2.4 wt %, with an overall average from all three samples of 1.8 wt % of Li (Fig. 3). Li is positively correlated with incompatible metals Rb, Mg, Be, Cs, and B (Fig. 3); weakly correlated with Fe and Y; and poorly correlated with Ti, Zr, Nb, Ba, La, and U (table S2). Most elements (including Li) overlap in composition between all three analyzed samples, although overall LNC-001 has lower B than LNC-072 and WLC-125 at any given Li concentration (table S2). Nonclay minerals (including feldspar, pyrite, and calcite) contain no measurable Li (Fig. 3). Semi-quantitative energy dispersive spectroscopy concentration data collected on samples LNC-072 and WLC-125 confirm that the clay contains abundant K, F, Mg, and Si and minor concentrations of Fe and Al, consistent with the ideal formula for tainiolite [KMg_2_LiSi_4_O_10_(OH,F)_2_] (table S3).

## DISCUSSION

The most abundant Li-bearing mineral in lacustrine rocks at McDermitt caldera is magnesian smectite, occurring throughout the whole caldera (Fig. 2) and averaging ~5100 ppm of Li from clay concentrates. This value is within the range measured on smectite clay concentrates at other lithium deposits (~3400–6800 ppm of Li) (Table 1). In the southern portion of McDermitt caldera near Thacker Pass, the sediments also contain Li-bearing illite with ~12,100 ppm of Li (in clay concentrates). The in situ analyses reveal that illites contain much higher Li, averaging ~18,000 ppm of Li (Fig. 3). This is approximately double the concentration of the smectites at Thacker Pass, elsewhere in McDermitt caldera, and at other smectite-hosted lithium deposits globally. The observed Li:Rb trend in the illites follows a linear enrichment trend (Fig. 3) from Thacker Pass smectite clay concentrates (*14*).

The Li-rich illite zone has only been identified in the southern half of the caldera at Thacker Pass (*10*) and immediately to the north in the Montana Mountains. Li-illite has not been identified in the northern part of the caldera in Oregon, where mining companies drilled through the caldera lake sediments into the underlying intracaldera tuff and never encountered assay values greater than ~3500 ppm of Li (*11*, *29*). The localization of the illitization around the resurgence-related faults indicates that extreme enrichment of Li in illites was associated with a spatially constrained hydrothermal event that post-dated the sedimentary and diagenetic processes responsible for the formation of Li-smectites across the whole caldera lake.

### Genetic model of Thacker Pass illite

#### Neoformation of Li-rich magnesian smectite

**Fig. 4.**
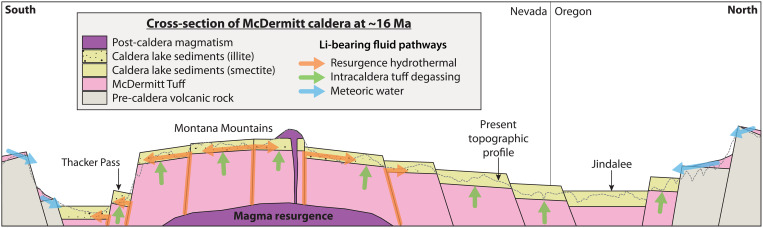
South-north schematic cross section of McDermitt caldera at the time of magmatic resurgence (~16 Ma ago). The present topographic profile is shown for reference as a dashed line with 12× vertical exaggeration. Resurgent uplift was centered around the Montana Mountains with only minor volumes of volcanism. Pathways of Li-bearing fluids into the system are indicated by colored arrows: leaching of outflow tuff by meteoric waters (blue), degassing of intracaldera tuff (green), and hydrothermal fluids associated with magmatic resurgence (orange). Li-bearing fluids associated with degassing of intracaldera tuff and leaching of outflow tuff supplied Li for smectite mineralization in the whole caldera. The fluids associated with magmatic resurgence, confined to the southern half of McDermitt caldera at Thacker Pass and the Montana Mountains, ascended from depth in intracaldera tuff along resurgence fractures, and spread laterally in the caldera lake sediments via microfractures and permeable tephra and carbonate layers, causing illitization and extreme Li enrichment in claystones, up to ~2.4 wt % of Li.

Before illitization, Li-rich magnesian smectite clays in the paleo McDermitt lake sediments precipitated (neoformed) due to reactions of clasts of volcanic glass with aqueous solutions in the highly alkaline and basic closed basin. A high-pH lacustrine setting is supported by the presence of carbonate beds and calcite nodules coeval with the smectite (*8*), which are notably similar in size and appearance to carbonates and spherulitic calcite nodules coeval with neoformed magnesian smectites in other alkaline lacustrine settings (*30*–*32*). In these high-pH (>9.5) analogs (*33*–*37*), including the modern East African Rift lakes (*38*–*41*), end-member Mg-smectite is typically formed [stevensite; Mg_3_Si_4_O_10_(OH)_2_]. Under the solute-rich and silica-saturated saline conditions favorable for the precipitation of stevensite, nanoparticles coalesce to form poorly crystalline gels, which, upon dehydration, become ordered and form stevensite sheets (*34*, *35*, *42*). In the McDermitt paleo-lake, a Li-rich Mg-smectite neoformed preferentially over pure stevensite because the lake water contained elevated activities of Li^+^, Rb^+^, F^−^, and other solutes due to the relatively high enrichment of these elements in the McDermitt source rhyolitic magma (*7*). Following the main eruption, the solutes were leached from outflow rhyolitic ignimbrite glass and other volcanic rock (*43*) during immediately post-eruptive cooling (*44*) and from the hot, thickly ponded intracaldera tuff during sustained devitrification and degassing (Fig. 4) (*9*, *45*). The McDermitt paleo lake served as a catchment basin for these solute- and silica-rich fluids, which became further concentrated to levels required for smectite saturation and precipitation (*35*) during evaporation in the closed caldera lake system.

Smectite neoformation is preferred over a diagenetic (*8*) or detrital model because the clays have high Mg/Al consistent with authigenic neoformation (*41*, *46*). In addition, little evidence exists in thin section or hand sample for diagenetic alteration (transformation) of glassy tuffaceous material to smectite in samples from drill cores other than minor bentonized tephra, only evident in the upper portion of the sedimentary section (*9*). Elsewhere in the deposit, finely laminated (sub-centimeter) claystone lithologies dominate and have sharp lithological contacts with interbedded tephra that are not altered to clay. If the clay formed through diagenetic alteration of glass, then sharp lithological contrasts between tephra and clay beds would not remain and clay-altered glass shards would be obvious in petrographic observations.

#### Hydrothermal alteration of smectite to illite during caldera resurgence

We interpret the ~40-m-thick horizon of Li-rich illite within the lacustrine sequence at Thacker Pass to represent the influx of Li-bearing hydrothermal fluids associated with caldera resurgence. This sedimentary interval is extremely enriched in Li (up to ~2.4 wt % of Li in situ; Fig. 3) and other trace elements common in hydrothermal fluids (e.g., Rb, Cs, Mo, and K) (*8*, *9*). Similar enrichments are observed at active post-caldera hydrothermal systems at vents on the Yellowstone caldera lake floor (*47*, *48*) and nearby geothermal systems (*49*), indicating the interaction of meteoric water with a deeper and hotter hydrothermal fluid with likely magmatic hydrothermal components.

The ca. 16.4 to 16.1 Ma ago resurgence (*8*) resulted in the uplift of the Montana Mountains and the formation of intracaldera fractures and faults focused just north of Thacker Pass along the western caldera margin (Figs. 2 and 4) (*20*). These fractures served as the conduits for ascending hydrothermal fluids exsolving from post-caldera subsurface resurgent magmas along the western ring fracture (*20*). Here, the fluids formed the Moonlight, Horse Creek, and Big Bend Spring deposits (Fig. 2) and are strongly enriched in U, Zr, and other metals commonly associated with epithermal precious metal deposits (As and Sb) (*9*, *50*). This distinct U- and Zr-rich resurgence mineralization formed 16.32 ± 0.10 Ma ago at temperatures around 300°C inferred from fluid inclusion experiments (*5*, *8*, *20*) and is considered unrelated to the relatively cooler ~200°C Hg (±U, Ga) mineralization at the Bretz, Opalite, McDermitt, and Cordero mines (Fig. 2) located >20 km away in the northern ring fracture zone of the caldera (*8*, *20*, *51*, *52*).

Although the resurgence-related hydrothermal fluids were initially moderately enriched in Li (*7*), as is commonly observed in post-caldera magmas in other F-rich caldera systems (*53*–*57*), most of the Li enrichment in the altering fluids likely occurred in the epithermal environment (*58*). As the initially hot (>300°C) mineralizing fluids cooled and ascended into the volcaniclastic intracaldera sedimentary section, they interacted with lacustrine-derived fluids and leached Li from Li-rich sediments proximal to the center of resurgence (Fig. 4). The resulting Li-bearing alkali-chloride-fluoride-carbonate fluids then migrated laterally away from the topographically high resurgence source (Fig. 4) in response to fluid pressure and gravity gradients (*58*, *59*) through carrier beds of enhanced permeability such as tephra and carbonate layers. The laterally migrating fluids produced primary carbonate dissolution and volume loss associated with deformation of the finely laminated sediments as evidenced by microfractures and folding observed at microscopic and megascopic scales (*9*) ~10 to 30 m above the top of the intracaldera ignimbrite (Fig. 2). The fluids presumably migrated under moderately low-pH and reducing conditions, like that measured in modern sublacustrine hydrothermal systems at Yellowstone (*60*). The lateral fluid flow caused widespread potassic alteration (Table 1) throughout the western portion of the caldera (*5*, *51*), resulting in decreased concentrations of metals (U and Zr) and epithermal metals (As and Sb) away from the source, a lateral or upward trace metal dispersal pattern observed in porphyry copper, epithermal, and other hydrothermal systems (*61*).

As the potassic hydrothermal fluid spread laterally, primary sedimentary carbonates dissolved and/or were replaced by fluorite (*9*), analcime was altered to K-feldspar (*8*), pyrite precipitated (*9*), and smectite began to dissolve and precipitate as Li-illite in a series of dissolution-precipitation reactions, leading to complicated intermediate and mixed-layer phases (*62*, *63*). Preliminary chemical reactions are presented in the Supplementary Materials using the documented compositions of the observed smectite and illite Thacker Pass (*14*). These reactions require addition of Li^+^, K^+^, and F^−^ by the hydrothermal solutions and loss of Al^+3^ and Na^+^.

In the hottest central portions of the potassic fluid front, the smectite was completely altered to Li-illite with extremely high concentrations of Li and K relative to the over- and underlying smectite intervals (Fig. 2 and Table 1). Above and below the zones of maximum alteration, smectite clays were only partially altered to illite, leading to mixed and interstratified illite/smectite zones (*8*, *9*, *14*) with clay (001) d-spacing intermediate between smectite and illite (Fig. 2). Multiple fluids were likely involved in the genesis of the most Li-rich zones, given petrographic observations of illite-pyrite veins crosscutting previously altered illite-rich sediments (*9*).

Illitization is correlative with an increase in concentration of Li, Rb (Fig. 3), and other epithermal metals from starting values in unaltered neoformed smectite to highest values in the purely illitic zone. In known Li-poor geothermal and epithermal systems the formation of illite or illite-smectite occurs at ~230° to 300°C and 180° to 230°C, respectively, compared to formation of smectites at temperatures substantially lower than 180°C (*58*, *59*, *64*, *65*). Therefore, the observed lateral and vertical clay mineral and chemical zonation is consistent with replacement of pre-existing neoformed Li-smectite by more Li-rich illite at higher temperatures by hydrothermal fluids rich in K, Li, Rb, and F.

Ages from tephra intercalated with caldera lake sediments indicate that sedimentation continued throughout resurgence until at least ca. 15.7 Ma ago (*8*), and an authigenic K-feldspar age from Thacker Pass of 14.87 ± 0.05 Ma ago (*8*) suggests that hydrothermal fluids circulated after sedimentation ceased. Basin and range faulting commenced ca. 12 Ma ago (*66*), reactivating the western caldera ring fracture and causing additional post-lacustrine uplift of the Montana Mountains. This additional uplift caused ~10° to 12° eastward tilting (*20*), leading to increased erosion of caldera lake sediments in the Montana Mountains, eroding much of the upper smectitic intervals and exposing the illite on the surface at Thacker Pass. Elsewhere in the caldera, the arcuate caldera faults protected most of the lake sediments from post-Basin and Range erosion, preserving them to the present day.

### Comparison to other volcano sedimentary lithium deposits

Other major known volcano sedimentary Li resources (e.g., Sonora, Mexico; Jadar, Serbia; Clayton Valley, Nevada; Fig. 1) occur in extensional basins unrelated to caldera structures but in close association with tuffaceous volcanic rock from which Li was leached (*7*, *43*). Li enrichment in sediments in these basins is primarily a function of the volume of Li-bearing tuffaceous source material in their watersheds and the degree of evapoconcentration of Li in saline waters in closed basins leading to Li-rich Mg-smectite authigenesis (*67*). At McDermitt caldera, the concentration of Li in the Mg-smectites is on par with other smectite deposits (Table 1), but the Li overall tonnage is considerably higher (table S1). This is due to the large volume (>500 km^3^) (*8*, *9*, *20*) of Li-rich (*7*) magma that was emplaced at high temperatures (~800°C) as intracaldera ignimbrite (*27*) and quickly overlain by the paleo McDermitt lake and attendant sediments. The resulting prolonged cooling and degassing interval was sufficient for sustained leaching of Li from glassy material (*44*) into the overlying lacustrine waters, a phenomenon only possible within unbreached calderas.

Initially hot (up to ~300°C) (*5*, *8*, *59*, *60*) intracaldera resurgent-related hydrothermal fluids lateral to the deposit provided the heat and Li mass necessary to alter the Mg-smectite to Li-illite at ~200° to 300°C and further drive up the concentration of Li in the high-grade zone. Li-illite is not present in the Li projects in Oregon adjacent to historic Hg mines (Fig 2) because the relatively lower-temperature (200°C or less) (*8*, *52*) hydrothermal fluids responsible for mineralization along the northern caldera ring fracture were not hot enough to alter the primary smectites to illites and had a smaller lateral footprint, as indicated by caldera-wide alteration mineral patterns (*51*). Volcano sedimentary systems at Hector, California and Rhyolite Ridge, Nevada (Fig. 1) are postulated to also have some degree of hydrothermal input (*14*). These systems tend to have higher Li grades than the Mg-smectites formed purely by evapoconcentration in Clayton valley (*14*, *67*) but contain considerably lower grades than the illites at Thacker Pass (Table 1). Secondary hydrothermal fluids can therefore alter smectite to mixed illite/smectite clays but to form pure Li-illite and exceptionally enriched ore with concentrations >1 wt %, the altering hydrothermal fluids need to be greater than ~200°C and voluminous. The one known exception to this is the Jadar deposit in Serbia (Fig. 1), with an indicated mineral resource of 0.7 MT of Li at 0.8 wt % (*68*). The primary ore mineral at this volcano sedimentary deposit is not a clay but rather the eponymous lithium borosilicate, jadarite [LiNaSiB_3_O_7_(OH)], which can fit up to 3.2 wt % of Li on an ideal stoichiometric basis (*69*). Ongoing research (*70*) indicates that this Li-rich mineral, to date only found in the Jadar deposit, formed during complex multistage diagenesis in a highly saline and alkaline evaporitic basin at temperatures well below 200°C.

### Implications for the exploration of volcano sedimentary lithium deposits

Most, if not all, known volcano sedimentary Li deposits occur in closed lacustrine basins (*8*, *14*, *68*) with relatively high rates of evaporation resulting in the neoformation of Li-bearing magnesian clays (and/or jadarite). Metallogenesis in a caldera setting is what distinguishes the Li-rich sediments of McDermitt caldera as the largest known Li accumulation in the world (*8*) and the ~1.8 wt % of Li Thacker Pass illite as the highest-grade known Li-bearing claystone globally. Exploration for volcano sedimentary deposits of similar tonnage and grade to McDermitt should therefore focus on preserved lacustrine sediments in voluminous caldera systems and not extensional basins.

Most calderas, however, are not prospective for Li. Li prospective calderas ideally require magmas that formed in an intracontinental setting with a peralkaline composition (*7*), as is observed at McDermitt (*25*) and most other volcano sedimentary systems (*14*). Hot and dry intracontinental peralkaline magmas are more likely to retain Li during magmatic differentiation (*7*) compared to hydrous and calc-alkaline equivalents such as the Caetano Caldera, Nevada and other Oligocene centers in the basin and range (*71*). This is because dry peralkaline magmas tend to contain less vapor and phenocrystic biotite into which Li may preferentially partition (*D*_Li_^vapor/melt^ ≈ 10; *D*_Li_^biotite/melt^ = 0.8 to 1.7) (*57*, *72*) and fractionate from the magma, resulting in relatively lower Li concentrations in the residual magma. A high initial Li concentration of the magma is essential to form a caldera-hosted Li deposit because in an eruption, as much as 45% of the Li could be lost to the surficial environment during and immediately following an eruption (*7*, *73*). Therefore, higher initial magmatic Li concentrations leads to higher Li concentrations in glassy intracaldera ignimbrite that can be leached during prolonged cooling and degassing (*9*, *73*).

Post-caldera resurgent doming is the final key ingredient to forming a massive caldera-hosted Li deposit. Ore deposits are commonly associated with resurgence magmatism globally (*74*, *75*), as the magmas provide both the hot hydrothermal fluids and ring and radial fractures associated with the doming provide conduits through which hydrothermal fluids can easily rise (Fig. 2). At McDermitt, the fluids exsolving from resurgent magma under Thacker Pass and ascending through the rigid fractures in intracaldera McDermitt Tuff were of sufficiently high initial temperature (>300°C) and Li activity (*5*, *8*, *20*) to alter the Mg-smectite to Li-illite once the fluids reached the intracaldera sediments and spread laterally (Fig. 4). Caldera systems with similar high-temperature, hydrothermally altered lacustrine sediments associated with intracaldera magmatic resurgence are therefore likely to serve as the best exploration targets to help meet increasing Li demand.

## MATERIALS AND METHODS

Three samples from dill core were prepared for in situ geochemical analysis. Table 2 describes their locations and depths.

**Table 2.** Locations and depths of samples analyzed in situ via SHRIMP-RG.

Hole ID	Latitude	Longitude	Sample depth (m)
LNC-001	41.695887	−118.073783	106.4
LNC-072	41.712627	−118.070432	55.2
WLC-125	41.708742	−118.068872	29.7

For sample LNC-001, a 6-cm-tall section of caldera lake sediment core containing lithium-bearing clays was embedded in epoxy to provide strength and then trimmed on diamond wafering saw to approximately 5 mm by 5 mm by 60 mm. To fit into a 25.4-mm ion probe mount, the sample was cut into four smaller pieces and remounted in epoxy. The sample was carefully polished using 9-μm sandpaper, 3- and 1-μm diamond grit film to minimize topography, and 1-μm diamond polishing pad for the final polish.

For samples LNC-072 and WLC-125, thin sections of core containing lithium-bearing clays were mounted in epoxy to fit into a 25.4-mm ion probe mount. The mount was then carefully polished using 9-μm sandpaper, 3- and 1-μm diamond grit film to minimize topography, and 1-μm diamond polishing pad for the final polish.

In situ trace element analyses of clay layers (table S2) were performed on the Stanford U.S. Geological Survey SHRIMP-RG at Stanford University during two separate sessions in 2018 (LNC-001) and 2019 (LNC-072 and WLC-125). Secondary ions, accelerated at 10 kV, were sputtered from the target spot using an O_2_^−^ primary ion beam with an intensity varying from 0.3 to 0.7 nA. The primary ion beam spot had a diameter between 12 and 16 μm and a depth of ~1 to 2 μm to avoid detrital sedimentary and volcanic grains. The acquisition routine included analysis of ^6^Li^+^, ^11^B^+^, ^30^Si^+^, ^35^Cl^+^, ^85^Rb^+^, ^89^Y^+^, ^90^Zr^+^, and ^139^La^+^. The 2018 session also included measurement of ^24^Mg^16^O^+^, ^47^Ti^+^, and ^54^Fe^+^, and the 2019 session also included ^9^Be^+^, ^93^Nb^+^, ^133^Cs^+^, and ^238^U^16^O^+^. Lithium was the primary focus of these measurements; other elements were measured to monitor if the spot overlapped with nonclay material (e.g., Fe, Y, and La) and to evaluate if other fluid-related trace elements correlated with Li-enrichment. Optical and chemical inspection of each spot was performed to ascertain if the beam completely overlapped the clay or not. Analyses for which the beam intersected nonclay material (epoxy, gaps, and other minerals) are not included in this study because we only focused on the concentration of the clay itself.

Trace element concentrations were standardized using natural glass standards RLS-132, Macusani (*76*), GSD-1G (*77*), and GOR-132G (*78*). The goal was to include natural glass standards with elevated Li content; the highest reference material is Macusani glass (3400 ppm of Li) (*76*). Standard reference material NIST-611 (*79*) was also measured. The second tab in table S2 lists the standards used for each calibration curve in both sessions.

Analyses were performed using a single scan by peak-hopping through the mass table, and each mass is measured on a single EPT discrete dynode electron multiplier operated in pulse counting mode. Count times for ^6^Li was 10 s; all other trace element measurement count times ranged from 4 to 15 s to optimize counting statistics for each isotope, except for Ba in the 2019 session, which was measured for 0.1 s only to identify spots overlapping feldspar (concentrations note reported). The background for the electron multiplier is very low (<0.05 cps) and is statistically insignificant for the trace elements reported in this study.

Measurements were performed at mass resolutions of M/ΔM = ~10,500 (10% peak height measured on ^30^Si). Concentrations were calculated using the same approach as (*7*). Count rates of each element were ratioed to ^30^Si to account for any primary current drift, and derived ratios for the unknowns are compared to an average of those for the standards to determine concentrations. Calibration curves for the 2018 and 2019 SHRIMP-RG sessions were plotted using measured ratios and published concentrations for standard glasses (table S2). For each element, calibration curves were calculated using the average and SD of values measured for each standard glass.

For lithium, the calibration curve included only natural glass standards (excluding NIST-611) and was not fixed through the origin. The calibration line was fit through the origin for some elements if the concentration was low in the standards and the unknowns (e.g., Y and Zr) or limited standard compositional data was available. Thus, some of the calculated values for nonclay yield negative values because the line does not go through the origin in an effort to make the clay values as accurate as possible. Those values that are negative or close to zero were omitted because they were not clay and not the focus of the study. Data from synthetic NIST-611 glass were only included in the calibration if there was insufficient published values for natural glass standards to produce a calibration curve. We considered the natural glasses to be preferable because they are most similar in composition to unknowns, and the NIST synthetic glasses often define a slightly different calibration trend from natural sample calibrations, which we attribute to matrix effects.

Analytical errors are derived from the 68% confidence bands about the linear fit of the calibration curve. Although the Li concentrations of the McDermitt clays are ~3 to 5 times higher than Macusani reference material (3400 ppm of Li), the response of the detector is typically linear over this range of compositions based on results from previous studies (*7*). In addition, the reported uncertainty derived from the confidence bands increases with increasing Li concentration, which likely accounts for any change in response of the detector at very high count rates.

## Supplementary Materials

### This PDF file includes:

Supplementary Text Legends for tables S1 to S3 References
