# Supplementary material for: Hydrothermal enrichment of lithium in intracaldera illite-bearing claystones
Source: Sci Adv. 2023 Aug 30;9(35):eadh8183. doi: 10.1126/sciadv.adh8183 (PMC10468124; doi:10.1126/sciadv.adh8183)
Supplement: Supplementary file 1 — Supplementary Text Legends for tables S1 to S3 References [file sciadv.adh8183_sm.pdf]

Science Advances
AAAS

# Supplementary Materials for

## Hydrothermal enrichment of lithium in intracaldera illite-bearing claystones

Thomas R. Benson *et al.*

Corresponding author: Thomas R. Benson, thomasrbenson@gmail.com

*Sci. Adv.* **9**, eadh8183 (2023)
DOI: 10.1126/sciadv.adh8183

**The PDF file includes:**

Supplementary Text
Legends for tables S1 to S3
References

**Other Supplementary Material for this manuscript includes the following:**

Tables S1 to S3

## Supplementary Materials

Hectorite to Tainiolite Reaction

Li-F hectorite reaction to Li-F tainiolite, using approximate average compositions from *(14)*. Note that we have ignored Ti, Fe in the octahedral site, and Al in tetrahedral site. For reaction (1), the apparent OH:F is 1:3 in hectorite, and K ~ Na in the interlayer site, minor Al in the Octahedral site. Na:K ratios, and Na, K, Fe, Al contents are quite variable.

The reactions below are written as aqueous fluoride species complexation, but both chloride species complexation, or bare ion species are equally viable (i.e., KF, KCl, $K^+$). See Reactions 3 and 4 for the equivalent reactions as bare ions.

(1) $(K_{0.2}Na_{0.25})^{XII}(Al_{0.4}Mg_2Li_{0.35})^{VI}Si_4O_{10}((OH)_1F_1)) + 0.8KF + 0.65LiF + 0.5HF$

$= (K)^{XII}(Mg_2Li)^{VI}Si_4O_{10}((OH)_{0.5}F_{1.5}) + 0.4AlF_3 + 0.25NaF + 0.5H_2O$

$K_{equilibrium} = (aAlF_3)^{0.4}*(aNaF)^{0.25}/\{(aKF)^{0.8}*(aLiF)^{0.65}(aHF)^{0.5}\}$

Added 0.65 ($Li^+$).

Added 1.95 ($F^-$), lost 1.45(F-). Delta = +0.5 $F^-$

Addition ratio is Li:F ~0.65:0.5 or 4:3.

***************************

As in (1), Li-F hectorite reaction to tainiolite, using approximate average compositions from *(14)*, but modified to include (OH) site to be 100% filled with F.

(2) $(K_{0.2}Na_{0.25})^{XII}(Al_{0.4}Mg_2Li_{0.35})^{VI}Si_4O_{10}((OH)_1F_1)) + 0.8KF + 0.65LiF + 1HF$

$= (K)^{XII}(Mg_2Li)^{VI}Si_4O_{10}(F_2) + 0.4AlF_3 + 0.25NaF + H_2O$

$K_{equilibrium} = (a\ AlF_3)^{0.4}*(aNaF)^{0.25}/\{(aKF)^{0.8}*(aLiF)^{0.65}(aHF)\}$

Added 0.65 (Li+).

Added 2.45 ($F^-$), lost 1.45(F-). Delta = +1 $F^-$

Addition ratio is Li:F ~0.65:1 or 2:3 (see plot)

***************************

(3) $(K_{0.2}Na_{0.25})^{XII}(Al_{0.4}Mg_2Li_{0.35})^{VI}Si_4O_{10}((OH)_1F_1)) + 0.8K^+ + 0.65Li^+ + 0.5H^+ + 0.5F^-$

$= (K)^{XII}(Mg_2Li)^{VI}Si_4O_{10}((OH)_{0.5}F_{1.5}) + 0.4Al^{+3} + 0.25Na^+ + 0.5H_2O$

$K_{equilibrium} = (aAl^{+3})^{0.4}*(aNa^+)^{0.25}/\{(aK^+)^{0.8}*(aLi^+)^{0.65}*(aH^+)^{0.5}*(aF^-)^{0.5}\}$

Added $0.65Li^+$, $0.8\ K^+$, $0.5\ H^+$

Added 1.95 ($F^-$), lost 1.45(F-). Delta = +0.5 $F^-$

Addition ratio is Li:F ~0.65:0.5 or 4:3.

Lost, $0.4Al^{+3}$, $0.25\ Na^+$

***************************

As in (1), Li-F hectorite reaction to tainiolite, using approximate average compositions from *(14)*, but modified to include (OH) site to be 100% filled with F.

(4) $(K_{0.2}Na_{0.25})^{XII}(Al_{0.4}Mg_2Li_{0.35})^{VI}Si_4O_{10}((OH)_1F_1)) + 0.8KF + 0.65LiF + 1HF$

$= (K)^{XII}(Mg_2Li)^{VI}Si_4O_{10}(F_2) + 0.4AlF_3 + 0.25NaF + H_2O$

$K_{equilibrium} = (aAl^{+3})^{0.4}*(aNa^+)^{0.25}/\{(aK^+)^{0.8}*(aLi^+)^{0.65}*(aH^+)*(aF^-)\}$

Added $0.65Li^+$, $0.8\ K^+$, $1H^+$

Added +1 $F^-$

Addition ratio is Li:F ~0.65:1 or 2:3 (see plot)

Lost, $0.4Al^{+3}$, $0.25\ Na^+$

**Additional Supplementary Files**

Table S1 Global Lithium Resources Database (*xlsx)

Table S2 SHRIMP-RG Results (*xlsx)

Table S3 EDS Data (*xlsx)

## REFERENCES AND NOTES


1. Benchmark Mineral Intelligence, “Lithium Forecast Q4 2022”; www.benchmarkminerals.com/forecasts/

2. U.S. Geological Survey, (USGS), “Mineral commodity summaries 2023” (USGS, 2023); doi: 10.3133/mcs2023.

3. “FACT SHEET: Securing a made in America supply chain for critical minerals,” *The White House*, 22 February 2022; www.whitehouse.gov/briefing-room/statements-releases/2022/02/22/fact-sheet-securing-a-made-in-america-supply-chain-for-critical-minerals/

4. R. K. Glanzman, J. H. McCarthy Jr., J. J. Rytuba, Lithium in the McDermitt Caldera, Nevada and Oregon. *Energy* **3**, 347–353 (1978).

5. J. J. Rytuba, R. K. Glanzman, Relation of Mercury, Uranium, and Lithium deposits to the McDermitt Caldera Complex, Nevada-Oregon (U.S. Geological Survey, Open-File Report 78-926, 1978).

6. I. E. Odom, Hectorite Deposits in the McDermitt Caldera of Nevada, paper presented at the Society for Mining, Metallurgy, and Engineering Annual Meeting, Phoenix, AZ, 24 to 27 February 1992.

7. T. R. Benson, M. A. Coble, J. J. Rytuba, G. A. Mahood, Lithium enrichment in intracontinental rhyolite magmas leads to Li deposits in caldera basins. *Nat. Commun.* **8**, 270 (2017).

8. S. B. Castor, C. D. Henry, Lithium-rich claystone in the McDermitt Caldera, Nevada, USA: Geologic, mineralogical, and geochemical characteristics and possible origin. *Minerals* **10**, 68 (2020).

9. J. T. Ingraffia, M. W. Ressel, T. R. Benson, Thacker Pass lithium clay deposit, McDermitt caldera, north-central Nevada: Devitrification of McDermitt Tuff as the main lithium source (Geological Society of Nevada Special Publication, 2020), pp. 395–410.

10. Lithium Americas Corporation, “Lithium Americas Provides General Motors Transaction Details and Update on Construction Plan for Thacker Pass” (2023); www.lithiumamericas.com/news/lithium-americas-provides-general-motors-transaction-details-and-update-on-construction-plan-for-thacker-pass

11. Jindalee Resources Limited, "Mineral Resource at McDermitt Increases to 21.5 Mt LCE, Now the Largest Lithium Deposit in the US" (2023); www.jindalee.net/site/pdf/a01eecf1-e9c2-45de-af32-c862da0f08ce/Resource-at-McDermitt-increases-to-215-Mt-LCE.pdf

12. S. E. Kesler, P. W. Gruber, P. A. Medina, G. A. Keoleian, M. P. Everson, T. J. Wallington, Global lithium resources: Relative importance of pegmatite, brine, and other deposits. *Ore Geol. Rev.* **48**, 55–69 (2012).

13. K. K. Rankama, T. G. Sahama, *Geochemistry* (Chicago Univ. Press, 1951).

14. C. Morissette, "The impact of geological environment on the lithium concentration and structural composition of hectorite clays," thesis, University of Nevada-Reno, Reno, NV (2012).

15. T. L. Barry, S. P. Kelley, S. P. Reidel, V. E. Camp, S. Self, N. A. Jarboe, R. A. Duncan, P. R. Renne, Eruption chronology of the Columbia River Basalt Group. *Geol. Soc. Am. Spec. Pap.* **497**, 45–66 (2013).

16. G. A. Mahood, T. R. Benson, Using $^{40}Ar/^{39}Ar$ ages of intercalated silicic tuffs to date flood basalts: Precise ages for Steens Basalt Member of the Columbia River Basalt Group. *Earth Planet. Sci. Lett.* **459**, 340–351 (2017).

17. J. Kasbohm, B. Schoene, Rapid eruption of the Columbia River flood basalt and correlation with the mid-Miocene climate optimum. *Sci. Adv.* **4**, eaat8223 (2018).

18. V. E. Camp, R. E. Wells, The case for a long-lived and robust yellowstone hotspot. *GSA Today* **31**, 4–10 (2020).

19. M. A. Coble, G. A. Mahood, Geology of the High Rock caldera complex, northwest Nevada, and implications for intense rhyolitic volcanism associated with flood basalt magmatism and the initiation of the Snake River Plain–Yellowstone trend. *Geosphere* **12**, 58–113 (2016).

20. C. D. Henry, S. B. Castor, W. A. Starkel, B. S. Ellis, J. A. Wolff, J. A. Laravie, W. C. Mcintosh, M. T. Heizler, Geology and evolution of the McDermitt caldera, northern Nevada and southeastern Oregon, western USA. *Geosphere* **13**, 1066–1112 (2017).

21. R. R. Compton, Contact Metamorphism in the Santa Rosa range, Nevada. *Geol. Soc. Am. Bull.* **71**, 1383–1416 (1960).

22. D. B. Burke, N. J. Silberling, The Auld Land Syne Group, of Late Triassic and Jurassic (?) age, north-central Nevada (U.S. Geological Survey Bulletin 1394-E, 1973), pp. 1–14.

23. S. J. Wyld, J. E. Wright, New evidence for Cretaceous strike-slip faulting in the United States Cordillera and implications for terrane-displacement, deformation patterns, and plutonism. *Am. J. Sci.* **301**, 150–181 (2001).

24. M. E. Brueseke, W. K. Hart, Geology and petrology of the mid-Miocene Santa Rosa-Calico volcanic field, northern Nevada. *Nevada Bureau of Mines and Geology* **113**, 1–44 (2008).

25. J. J. Rytuba, E. H. McKee, Peralkaline ash flow tuffs and calderas of the McDermitt volcanic field, Southeast Oregon and North Central Nevada. *J. Geophys. Res.* **89**, 8616–8628 (1984).

26. T. R. Benson, G.A. Mahood, M. Grove, Geology and $^{40}Ar/^{39}Ar$ geochronology of the Middle Miocene McDermitt Volcanic Field, Oregon and Nevada: Silicic volcanism associated with propagating flood basalt dikes at initiation of the Yellowstone hotspot. *Geol. Soc. Am. Bull.* **129**, 1027–1051 (2017).

27. H. R. Hargrove, M. F. Sheridan, Welded tuffs deformed into megarheomorphic folds during collapse of the McDermitt Caldera, Nevada-Oregon. *J. Geophys. Res.* **89**, 8629–8638 (1984).

28. R. Ehsani, L. Fourie, A. Hutson, D. Peldiak, R. Spiering, J. Young, K. Armstrong, Technical Report on the Pre-Feasibility Study for the Thacker Pass Project, Humboldt County, Nevada, USA (Lihium Americas Corp., 2018); www.lithiumamericas.com/_resources/pdf/investors/technical-reports/thacker-pass/Technical-Report-Thacker-Pass.pdf

29. Aurora Energy Metals, "Lithium Zone Expanded Significantly, Open in All Directions" (2023); www.investi.com.au/api/announcements/1ae/439dd9a0-b0c.pdf

30. V. P. Wright, Lacustrine carbonates in rift settings: the interaction of volcanic and microbial processes on carbonate deposition. *Geol. Soc. London Spec. Publ.* **370**, 39–47 (2012).

31. R. Mercedes-Martín, A. T. Brasier, M. Rogerson, J. J. G. Reijmer, H. Vonhof, M. Pedley, A depositional model for spherulitic carbonates associated with alkaline, volcanic lakes. *Mar. Pet. Geol.* **86**, 168–191 (2017).

32. F. Farias, P. Szatmari, A. Bahniuk, A. B. França, Evaporitic carbonates in the pre-salt of Santos Basin – Genesis and tectonic implications. *Mar. Pet. Geol.* **105**, 251–272 (2019).

33. J. Calvo, M. Blanc-Valleron, J. Rodriguez-Arandia, J. Rouchy, M. Sanz, Authigenic clay minerals in continental evaporitic environments (Special Publications of the International Association of Sedimentologists, 1999), vol. 27, pp. 129–151.

34. N. J. Tosca, A. L. Masterson, Chemical controls on incipient Mg-silicate crystallization at 25°C: Implications for early and late diagenesis: Implications for early and late diagenesis. *Clay Miner.* **49**, 165–194 (2014).

35. N. J. Tosca, V. P. Wright, The Formation and Diagenesis of Mg-Clay Minerals in Lacustrine Carbonate Reservoirs, paper presented at the AAPG Annual Convention and Exhibition, Houston, Texas, 6 to 9 April 2014.

36. J. P. Gomes, R. B. Bunevich, L. R. Tedeschi, M. E. Tucker, F. F. Whitaker, Facies classification and patterns of lacustrine carbonate deposition of the Barra Velha Formation, Santos Basin, Brazilian Pre-salt. *Mar. Pet. Geol.* **113**, 104176 (2020).

37. P. R. A. Netto, M. Pozo, M. D. da Silva, A. S. Mexias, M. E. B. Gomes, L. Borghi, A. M. Rios-Netto, Authigenic Mg-clay assemblages in the Barra Velha Formation (Upper Cretaceous) from Santos Basin (Brazil): The role of syngenetic and diagenetic process. *Appl. Clay Sci.* **216**, 106339 (2022)

38. J. Y. Gac, A. Droubi, B. Fritz, Y. Tardy, Geochemical behaviour of silica and magnesium during the evaporation of waters in Chad. *Chem. Geol.* **19**, 215–228 (1977).

39. F. Darragi, Y. Tardy, Authigenic trioctahedral smectites controlling pH, alkalinity, silica and magnesium concentrations in alkaline lakes. *Chem. Geol.* **63**, 59–72 (1987).

40. T. E. Cerling, Chemistry of closed basin lake waters: A comparison between African Rift Valley and some central North American rivers and lakes, *in Global Geological Record of Lake Basins 1*, E. Gierlowski-Kordesch, K. Kelts, Eds. (Cambridge Univ. Press, 1994), pp. 29–30.

41. V. C. Hover, G. M. Ashley, Geochemical signatures of paleodepositional and diagenetic environments: A STEM/AEM study of authigenic clay minerals from an arid rift basin, Olduvai Gorge, Tanzania. *Clays Clay Miner.* **51**, 231–251 (2003).

42. C. de S. Buey, M. S. Barrios, E. G. Romero, M. D. Montoya, Mg-rich smectite “precursor” phase in the Tagus Basin, Spain. *Clays Clay Miner.* ***48***, 366–373 (2000).

43. J. G. Price, P. J. Lechler, M. B. Lear, T. F. Giles, Possible volcanic source of lithium in Brines in Clayton Valley, Nevada in *Geology and Ore Deposits 2000: The Great Basin and Beyond Proceedings* (2000), vol. 1, pp. 241–248.

44. B. S. Ellis, D. Szymanowski, C. Harris, P. M. E. Tollan, J. Neukampf, M. Guillong, E. A. Cortes-Calderon, O. Bachmann, Evaluating the potential of rhyolitic glass as a lithium source for brine deposits. *Econ. Geol.* **117**, 91–105 (2022).

45. N. C. Sturchio, K. Muehlenbachs, M. G. Seitz, Element redistribution during hydrothermal alteration of rhyolite in an active geothermal system: Yellowstone drill cores Y-7 and Y-8. *Geochim. Cosmochim. Acta* **50**, 1619–1631 (1986).

46. J. L. Bentz, R. C. Peterson, The formation of clay minerals in the mudflats of Bolivian Salars. *Clays Clay Miner.* **68**, 115–134 (2020).

47. L. S. Balistrieri, W. C. Shanks III, R. L. Cuhel, C. Aguilar, J. V. Klump, The influence of sublacustrine hydrothermal vents on the geochemistry of Yellowstone Lake, in *Integrated Geoscience Studies in the Greater Yellowstone Area: Volcanic, Tectonic, and Hydrothermal Processes in the Yellowstone Geoecosystem*, L.A. Morgan Ed. (U.S. Geological Survey Professional Paper 1717, 2007), pp. 169–199.

48. W. C. Shanks III, J. C. Alt, L. A. Morgan, Geochemistry of sublacustrine hydrothermal deposits in Yellowstone lake-hydrothermal reactions, stable-isotope systematics, sinter deposition, and spire

formation. in *Integrated Geoscience Studies in the Greater Yellowstone Area: Volcanic, Tectonic, and Hydrothermal Processes in the Yellowstone Geoecosystem*, L.A. Morgan Ed. (U.S. Geological Survey Professional Paper 1717, 2007), pp. 201–234.

49. K. E. Bargar, M. H. Beeson, R. O. Fournier, P. L. Muffler, Present-day deposition from thermal waters of lepidolite in Yellowstone National Park. *Am. Mineral.* **58**, 901–904 (1973).

50. S. B. Castor, C. D. Henry, Geology, geochemistry, and origin of volcanic rock-hosted uranium deposits in northwestern Nevada and southeastern Oregon, USA. *Ore Geol. Rev.* **16**, 1–40 (2000).

51. J. J. Rytuba, D. A. John, A. F. Foster, S. D. Ludington, B. Kotlyar, Hydrothermal Enrichment of Gallium in Zones of Advanced Argillic Alteration – Examples from the Paradise Peak and McDermitt Ore Deposits, Nevada, in *Contributions to Industrial-Minerals Research*, J. D. Bliss, R. R. Moyle, K. R. Long Eds. (U.S. Geological Survey Bulletin 2209-C, 2003), pp. 1–16.

52. M. J. Hetherington, E. S. Cheney, Origin of the opalite breccia at the McDermitt mercury mine, Nevada. *Econ. Geol.* **80**, 1981–1987 (1991).

53. C. K. Richardson, H. D. Holland, Fluorite deposition in hydrothermal systems. *Geochim. Cosmochim. Acta* **43**, 1327–1335 (1979).

54. C. N. Mercer, A. H. Hofstra, T. I. Todorov, J. Roberge, A. Burgisser, D. T. Adams, M. Cosca, Pre-eruptive conditions of the hideaway Park topaz rhyolite: Insights into metal source and evolution of magma parental to the Henderson porphyry molybdenum deposit, Colorado. *J. Petrol.* **56**, 645–679 (2015).

55. K. Breiter, J. Ďurišová, T. Hrstka, Z. Korbelová, M. Hložková Vaňková, M. Vašinová Galiová, V. Kanický, P. Rambousek, I. Knésl, P. Dobeš, M. Dosbaba, Assessment of magmatic vs. metasomatic processes in rare-metal granites: A case study of the Cínovec/Zinnwald Sn–W–Li deposit, Central Europe. *Lithos* **292-293**, 198–217 (2017).

56. J. M. Rosera, "*The origin of volatile-rich silicic magmas and implicaitons for crustal pre-conditioning of porphyry-style mineralization*," thesis, University of North Carolina–Chapel Hill (2020).

57. J. D. Webster, J. R. Holloway, R. L. Hervig, Partitioning of lithophile trace elements between $H_2O$ and $H_2O$ + $CO_2$ fluids and topaz rhyolite melt. *Econ. Geol.* **84**, 116–134 (1989).

58. J. H. Dilles, D. A. John, Porphyry and epithermal mineral deposits, *in Encyclopedia of Geology*, J. D. Bliss, R. R. Moyle, K. R. Long, Eds. (Elsevier, 2020), vol. 5, pp. 847–866.

59. D. A. John, R. G. Lee, G. N. Breit, J. H. Dilles, A. T. Calvert, L.J. Patrick Muffler, M. A. Clynne, Pleistocene hydrothermal activity on Brokeoff volcano and in the Maidu volcanic center, Lassen Peak area, northeast California: Evolution of magmatic-hydrothermal systems on stratovolcanoes. *Geosphere* **15**, 946–982 (2019).

60. C. Tan, A. P. G. Fowler, A. Tudor, W. E. Seyfreid Jr., Heat and mass transport in sublacustrine vents in Yellowstone Lake, Wyoming: In-situ chemical and temperature data documenting a dynamic hydrothermal system. *J. Volcanol. Geotherm. Res.* **405**, 107043 (2020).

61. S. Halley, J. H. Dilles, R. M. Tosdal, Footprints: Hydrothermal alteration and geochemical dispersion around porphyry copper deposits. *SEG Discov.* **100**, 1–17 (2015).

62. L. Ohazuruike, K. J. Lee, A comprehensive review on clay swelling and illitization of smectite in natural subsurface formations and engineered barrier systems. *Eng. Technol.* **55**, 1495–1506 (2023).

63. E. Ferrage, O.Vidal, R. Mosser-Ruck, M. Cathelineau, J. Cuadros, A reinvestigation of smectite illitization in experimental hydrothermal conditions: Results from X-ray diffraction and transmission electron microscopy. *Am. Mineral.*, **96**, 207–223 (2011).

64. A.G. Reyes, Petrology of Philippine geothermal systems and the application of alteration mineralogy to their assessment. *J. Volcanol. Geotherm. Res.* **43**, 279–309 (1990)

65. I. Chambefort, J. H. Dilles, Chemical vectoring in continental geothermal systems: Composition of altered rocks and illite as guides to magmatic degassing. *Geothermics* **110**, 102682 (2023).

66. J. P. Colgan, T. A. Dumitru, P. W. Reiners, J. L. Wooden, E. L. Miller, Cenozoic tectonic evolution of the basin and range province in Northwestern Nevada. *Am. J. Sci.* **306**, 616–654 (2006).

67. C. A. Gagnon, K. L. Butler, E. Gaviria, A. Terrazas, A. Gao, T. Bhattacharya, D. Boutt, L. A. Munk, D. E. Ibarra, Paleoclimate controls on lithium enrichment in Great Basin Pliocene – Pleistocene lacustrine clays. *Geol. Soc. Am. Bull.* https://doi.org/10.1130/B36572.1 (2023).

68. Rio Tinto, "Rio Tinto updates Ore Reserves and Mineral Resources at Jadar": News release dated 23 February 2022; https://www.riotinto.com/-/media/Content/Documents/Invest/Reserves-and-resources/2021/RT-Jadar-reserves-resources-2021.pdf?rev=e1220b9671424ad9b275dd6bdd2ed480.

69. C. J. Stanley, G.C. Jones, M. S. Rumsey, C. Blake, A. C. Roberts, J.A.R. Stirling, G.J.C. Carpenter, P. S. Whitfield, J. D. Grice, Y. Lepage, Jadarite, $LiNaSiB_3O_7(OH)$, a new mineral species from the Jadar Basin, Serbia. *Eur. J. Mineral.* **19**, 575–580 (2007).

70. F. Putzolu, R. N. Armstrong, J. Garcia, N. Hawkes, E. Nebel, A. Boyce, J. Najorka, R.J. Herrington, Li residency in alkaline paleo-lake systems: The peculiar case of the Jadar (Serbia) deposit. *Geol. Soc Am. Abstr Programs* **54**, 5 (2022).

71. K. E. Watts, D. A. John, J. P. Colgan, C. D. Henry, I. N. Bindeman, A. K. Schmitt, Probing the volcanic-plutonic connection and the genesis of crystal-rich rhyolite in a deeply dissected supervolcano in the Nevada Great Basin: Source of the late Eocene Caetano Tuff. *J. Petrol.* **57**, 1599–1644 (2016).

72. J. Icenhower, D. London, An experimental study of element partitioning among biotite, muscovite, and coexisting peraluminous silicic melt at 200 MPa ($H_2O$). *Am. Mineral.* **80**, 1229–1251 (1995).

73. A. H. Hofstra, T. I. Todorov, C. N. Mercer, D. T. Adams, E. E Marsh, Silicate melt inclusion evidence for extreme pre-eruptive enrichment and post-eruptive depletion of lithium in silicic volcanic rocks of the Western United States: Implications for the origin of lithium-rich brines. *Econ. Geol.* **108**, 1691–1701 (2013).

74. R. L. Smith, R. A. Bailey, Resurgent Cauldrons. *Memoirs* **116**, 613–662 (1968).

75. J. J. Rytuba, Evolution of volcanic and tectonic features in caldera settings and their importance in localization of ore deposits. *Econ. Geol.* **89**, 1687–1696 (1994).

76. R. MacDonald, R. L. Smith, J. E. Thomas, Chemistry of the Subalkalic Silicic Obsidians (U.S. Geological Survey Professional Paper, 1992), vol. 1523, p. 214.

77. K. P. Jochum, S. A. Wilson, W. Abouchami, M. Amini, J. Chmeleff, A. Eisenhauer, E. Hegner, L. M. Iaccheri, B. Kieffer, J. Krause, W. F. McDonough, R. Mertz-Kraus, I. Raczek, R. Rudnick, D. Scholz, G. Steinhofel, B. Stoll, A. Stracke, S. Tonarini, D. Weis, U. Weis, J. D.Woodhead, GSD-1G and MPI-ding reference glasses for in situ and bulk isotopic determination. *Geostand. Geoanal. Res.* **35**, 193–226 (2011).

78. K. P. Jochum, B. Stoll, K. Herwig, M. Willbold, A. W. Hofmann, M. Amini, S. Aarburg, W. Abouchami, E. Hellebrand, B. Mocek, I. Raczek, A. Stracke, O. Alard, C. Bouman, S. Becker, M. Dücking, H. Brätz, R. Klemd, D. de Bruin, D. Canil, D. Cornell, C. J. de Hoog, C. Dalpé, L. Danyushevsky, A. Eisenhauer, Y. Gao, J. E. Snow, N. Groschopf, D. Günther, C. Latkoczy, M. Guillong, E. H. Hauri, H. E. Höfer, Y. Lahaye, K. Horz, D. E. Jacob, S. A. Kasemann, A. J. R. Kent, T. Ludwig, T. Zack, P. R. D. Mason, A. Meixner, M. Rosner, K. Misawa, B. P. Nash, J. Pfänder, W. R. Premo, W. D. Sun, M. Tiepolo, R. Vannucci, T. Vennemann, D. Wayne, J. D. Woodhead, MPI-DING reference glasses for in situ microanalysis: New reference values for element concentrations and isotope ratios. *Geochem. Geophys. Geosyst.* **7**, Q02008 (2006).

79. K.P. Jochum, U. Weis, B. Stoll, D. Kuzmin, Q. Yang, I. Raczek, D. E. Jacob, A. Stracke, K. Birbaum, D. A. Frick, D. Günther, Determination of reference values for NIST SRM 610–617 glasses following ISO guidelines. *Geostand. Geoanal. Res.* **35**, 397–429 (2011).

80. P. W. Gruber, P. A. Medina, G. A. Keoleian, S. E. Kesler, M. P. Everson, T. J. Wallington, Global lithium availability. *J. Ind. Ecol.* **15**, 760–775 (2011).

81. S. G. Peters, T. V. V. King, T. J. Mack, M. P. Chornack, eds., and the U.S. Geological Survey Afghanistan Mineral Assessment Team, Summaries of important areas for mineral investment and production opportunities of nonfuel minerals in Afghanistan (U.S. Geological Survey Open-File Report 2011–1204, 2011), p. 1810.

82. H. Vikström, S. Davidsson, M. Höök, Lithium availability and future production outlooks. *Appl. Energy* **110**, 252–266 (2013).

83. W. B. Simmons, A. U. Falster, G. Freeman, The Plumbago North pegmatite, Maine, USA: A new potential lithium resource. *Miner. Deposita* **55**, 1505–1510 (2020).

84. T. R. Benson, Lithium enrichment in claystones of the mid-Miocene Barstow Formation, Mojave Desert, California, in *Mines of the Mojave: 2023 Desert Symposium Field Guide and Proceedings*, D. M. Miller, S. M. Rowland, Eds. (Desert Symposium, 2023), p. 161.

85. S. M. Aleksandrov, Skarn-Greisen deposits of the lost river and Mount ear Ore Field, Seward Peninsula, Alaska, United States. *Geochemistry Int.* **48**, 12, 1220–1236 (2010).

86. B. Gourcerol, E. Gloaguen, J. Melleton, J. Tuduri, J. X. Galiegue, Re-assessing the European lithium resource potential – A review of hard-rock resources and metallogeny. *Ore Geol. Rev.* **109**, 494–519 (2019).

87. H. Minnaar, H. F. J. Theart, The exploitability of pegmatite deposits in the lower Orange River area (Vioolsdrif - Henkries - Steinkopf). *South Afr. J. Geol.* **109**, 341–352 (2006).

88. M. B. Dias, W. E. Wilson, The Alto Ligonha pegmatites: Mozambique. *Mineral. Rec.* **31**, 459–497 (2000).

89. R. Barros, D. Kaeter, J. F. Menuge, T. Fegan, J. Harrop, Rare Element Enrichment in Lithium Pegmatite Exomorphic Halos and Implications for Exploration: Evidence from the Leinster Albite-Spodumene Pegmatite Belt, Southeast Ireland. *Minerals* **12**, 1–21 (2022).

90. R. S. Gheshlaghi, M. Ghorbani, A. A. Sepahi, R. Deevsalar, K. Nakashima, R. Shinjo, The origin of gem spodumene in the Hamadan Pegmatite, Alvand Plutonic Complex, western Iran. *Can. Mineral.* **60**, 249–266 (2022).

91. L. Raimbault, Composition of complex lepidolite-type granitic pegmatites and of constituent columbite-tantalite. Chedeville, Massif Central, France. *Can. Mineral.* **36**, 563–583 (1998).

92. F. Risacher, B. Fritz, Quaternary geochemical evolution of the salars of Uyuni and Coipasa, Central Altiplano, Bolivia. *Chem. Geol.* **90**, 211–231 (1991).

93. M. Á. Galliski, M. F. Márquez-Zavalía, E. Roda-Robles, E. A. von Quadt, The Li-Bearing Pegmatites from the Pampean Pegmatite Province, Argentina: Metallogenesis and Resources. *Minerals* **12**, 841 (2022).
